# Radiotherapy for Oligometastases and Oligo-Recurrence of Bone in Prostate Cancer

**DOI:** 10.1155/2012/541656

**Published:** 2012-09-09

**Authors:** Ken-ichi Tabata, Yuzuru Niibe, Takefumi Satoh, Hideyasu Tsumura, Masaomi Ikeda, Satoru Minamida, Tetsuo Fujita, Daisuke Ishii, Masatsugu Iwamura, Kazushige Hayakawa, Shiro Baba

**Affiliations:** ^1^Department of Urology, Kitasato University School of Medicine, 1-15-1 Kitasato, Minami-ku, Sagamihara, Kanagawa 252-0375, Japan; ^2^Department of Radiology and Radiation Oncology, Kitasato University School of Medicine, 1-15-1 Kitasato, Minami-ku, Sagamihara, Kanagawa 252-0375, Japan

## Abstract

*Purpose*. To retrospectively evaluate the clinical significance of radiotherapy for oligometastases of bone in prostate cancer (PCa). *Methods and Materials*. Between 2003 and 2008, 35 PCa patients with oligometastases of bone were treated with radiotherapy. *Results*. The median radiotherapy dose was 40 Gy. The 3-year overall survival rates for all patients, for patients that received a radiotherapy dose of ≥40 Gy (*n* = 21) and for those that received <40 Gy (*n* = 14), were 77.2%, 90.5%, and 50.0%, respectively. Fourteen out of 16 patients (87.5%) who had pain were improved 1 month after radiotherapy. The median duration of pain relief was 12 months. Pathological fracture and spinal cord compression (SCC) were not seen at the treated sites but developed at nonirradiated sites in three patients (8.6%) and in one patient (2.8%), respectively. Although the high-dose group (≥40 Gy) achieved better survival than the low-dose group (<40 Gy), it was not independent prognostic factor in multivariable analysis. *Conclusions*. Radiotherapy of bone oligometastases in PCa was effective for long-term pain relief. Pathological fracture and SCC were not seen at the treated sites. A larger clinical trial is warranted to study the actual benefit following radiotherapy for oligometastases of bone in PCa.

## 1. Introduction

 Patients with bone metastases from prostate cancer frequently experience skeletal morbidities as a result of their disease. Skeletal-related events (SREs), such as pathological fractures and spinal cord compression, are major causes of morbidity in patients with prostate cancer and may lead to other comorbidities including pain [1, 2].

 Soloway et al. [3] reported an analysis of survival in prostate cancer patients with bone metastases using a semiquantitative grading system based upon the extent of disease (EOD) on the bone scintigram. They concluded that the EOD on the scintigram correlated with survival. This study also demonstrated that the 2-year survival rate in prostate cancer patients with EOD I, defined as having fewer than six bone metastases on bone scan, was 94%. Thus the clinical course of prostate cancer patients with a small number of bone metastases is relatively long. Successful management of bone metastases during these periods is essential for reducing the skeletal complications and for maximizing patients' quality of life. Therefore, we must carefully manage metastatic bone disease from an early stage in prostate cancer.

 The aim of radiotherapy for metastatic bone disease is not only relief of bone pain but also healing and prevention of pathological fractures, with anticipated effects including improved mobility, function, and quality of life [4, 5]. In addition to these effects, the notion of oligometastases and oligo-recurrence has recently been proposed [6–9], with the suggestion that local therapy to a small number of gross metastatic sites and recurrences may result in prolonged survival or even cure [6–10]. The most favorable prognostic factor of oligometastases is the state of primary lesion, which means that oligometastatic patients with controlled primary lesions achieve significant better survival than those with active primary lesions [11, 12]. The notion of oligo-recurrence overcomes this problem. Oligo-recurrence is the state that cancer patients with one to several metastases or recurrences have controlled primary lesions. Niibe and Hayakawa proposed this notion as oligo-recurrence [9].

 The objective of this retrospective study was to evaluate the effect of radiotherapy on bone oligometastases and oligo-recurrence in patients with prostate cancer. We were also interested in the disease behavior in patients with bone oligometastases and oligo-recurrence.

## 2. Methods and Materials

 Between January 2003 and December 2008, 136 Japanese men diagnosed with prostate cancer with bone metastases received radiotherapy directed at the metastatic bone lesions at Kitasato University Hospital, Japan. Their medical records were evaluated retrospectively. Thirty-five of the patients had bone metastases of EOD I, referred to as oligometastases or oligo-recurrence of bone in this study. EOD I has been defined by Soloway et al. [3] as the presence of fewer than six bone metastases on bone scan, with each site being less than 50% the size of a vertebral body. Indications for radiation to metastatic bone sites in patients with EOD I prostate cancer were bone pain or spinal cord compression, pathological fracture, or prevention of SREs.

 We analyzed the overall survival and the effect of radiotherapy on pain relief and the incidence of SREs, including pathological fracture and spinal cord compression. Short-term pain relief was determined by comparing symptoms prior to radiotherapy to that 1 month after its completion. Pain relief response was classified as follows by taking the best point from the start of treatment: “response,” when pain decreased or the daily dosage of the analgesic was decreased; “no change,” when pain was unchanged and the dosage of the analgesic did not change; and “progressive disease,” when pain increased or the dosage of the analgesic was increased. 

 For long-term pain relief, the time to progression was defined as the interval between the initial date of radiotherapy and the date when increased pain or increased dosage of the analgesic was first documented after the best pain relief response at treated sites. 

 Local treatment for prostate cancer might affect overall survival we divide patients into oligo-recurrence group which has treated enough locally such as prostatectomy and oligometastases group which has not been treated with local therapy for the prostate.

 Overall survival was calculated as the time interval from the last day of radiotherapy for bone metastases to the time of death. Progression-free survival for bone pain was defined as the proportion of patients surviving with decreased pain from the onset of pain relief to pain relapse at a treated site. Patients were followed for a median of 36 months (range, 1–70 months) after radiotherapy. 

 Radiotherapy was performed using one port postero-anterior field for the middle thoracic spine/upper lumbar spine and two ports anteroposterior parallel opposed fields for the other spine, legs, and pelvic bone. The energy of radiotherapy was 6 or 10 MV X-rays.

 The survival rate was calculated using the Kaplan-Meier method. Differences in patient characteristics between the two groups were compared by chi-square test or Fisher exact test, as appropriate. Multivariable analysis was performed by employing the Cox proportional hazards regression model to examine the interaction between total radiotherapy dose (≥40 Gy versus <40 Gy) and other clinical variables and to estimate the independent prognostic effect of radiotherapy on survival by adjusting for confounding factors. Within the present study population, there were 11 deaths, which allow a maximum of two variables to be included in a multivariable regression model. Therefore all potential confounding factors of radiotherapy dose were reduced to one single composite characteristic by applying a propensity score [13]. The conventional *P*  value < 0.05 was used to determine the level of statistical significance. Analyses were performed with Stata version 11 for Windows (Stata, Chicago, IL, USA).

## 3. Results

Table 1 shows the baseline characteristics of the study population according to the total radiotherapy dose. In prior treatment to the primary site, radical prostatectomy was performed in 10 patients, and radiotherapy including conformal external beam radiotherapy (3DCRT) alone and high dose rate brachytherapy (HDR) in combination with 3DCRT (HDR/3DCRT) was performed in eight patients. These eighteen patients were to be in the state of oligo-recurrence. Other seventeen patients are called as oligometastases group in this study. All 35 patients received hormonal therapy. Nine patients received Zoledronic acid. There were significant differences in baseline serum prostate-specific antigen, Eastern Cooperative Oncology Group performance status (ECOG PS) and oligostatus between total radiotherapy doses of ≥40 Gy and <40 Gy (Table 1). 

 Treatment characteristics are given in Table 2. The median local radiotherapy dose was 40 Gy (range, 30–50 Gy) in 10–25 fractions. The median biologically effective dose (BED) was 67 Gy_3_ (range, 50–92 Gy_3_) if *α*/*β* of 3 was applied. The reasons for radiotherapy were pain relief in 16 patients (45.7%), prevention of SREs in 17 patients (48.6%), and spinal cord compression in 2 patients (5.7%).

**Figure 1:**
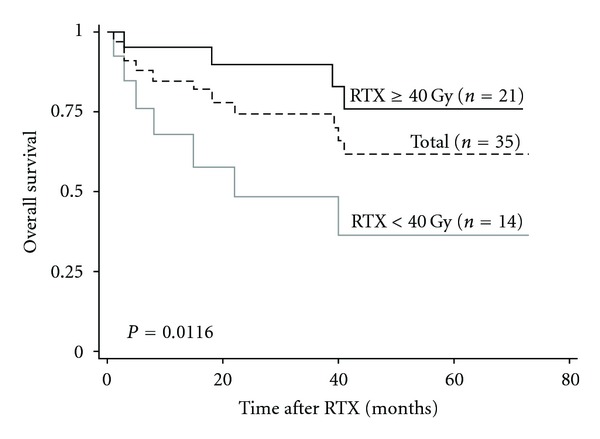
The overall survival curves for all patients (*n* = 35) and those that received a total radiotherapy dose of ≥40 Gy (*n* = 21) or <40 Gy (*n* = 14). RTX, radiotherapy.

**Figure 2:**
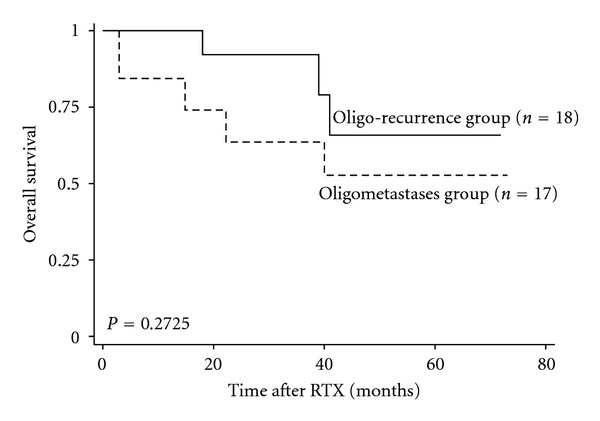
The overall survival curves for oligo-recurrence group (*n* = 18) and oligometastases group (*n* = 17). RTX, radiotherapy.

Figure 1 shows the overall survival curves after radiotherapy for metastatic bone disease. The 3-year overall survival rate for all patients was 77.2%. The overall survival rate of radiotherapy doses of >40 Gy and of <40 Gy was 90.5% and 50.0%, respectively (*P* = 0.0116). There is no significant difference between Oligo-recurrence group and Oligometastases group (Figure 2). A Cox proportional hazards model was applied to estimate the effect of radiotherapy dose on overall survival. The crude hazard ratio (HR) of high-dose group (≥40 Gy) compared with low-dose group (<40 Gy) was 0.231 (95% CI, 0.067–0.798; *P* = 0.021), which indicated that high-dose group decreased the hazard of death by four times that of low-dose group (Table 4). Then we performed multivariable analysis using propensity score to adjust the effect of receiving high-dose radiotherapy (≥40 Gy) given by other confounding variables including age, baseline PSA, ECOG PS, castration-resistant prostate cancer (CRPC), oligostatus into a single estimator. The results revealed that the HR of radiotherapy dose (≥40 Gy versus <40 Gy) changed to 0.630 (95% CI, 0.098–4.285; *P* = 0.637), which suggests that high-dose radiotherapy was not an independent risk factor for overall survival (Table 4).

**Figure 3:**
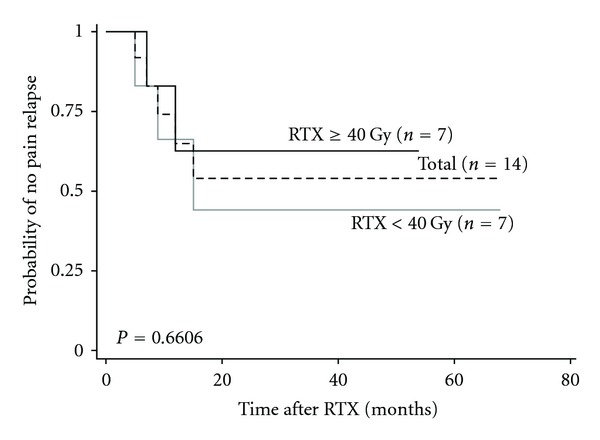
The progression-free survival curves for patients with bone pain who had pain relief response at 1 month after radiotherapy (*n* = 14) and with received total radiotherapy dose of ≥40 Gy (*n* = 7) and <40 Gy (*n* = 7). RTX, radiotherapy.

 The treatment outcomes are shown in Table 3. At 1 month after radiotherapy, 14 out of 16 patients (87.5%) with pain gained relief. Five of these patients (31.3%), however, experienced pain relapse in the treated sites.  Figure 3 shows the progression-free survival for bone pain. One-year progression-free survival was 64.8%, and the median duration of pain relief was 12 months (range, 5–68 months). Two patients had a relapse of bone pain within 1 year after radiotherapy in ≥40 Gy and <40 Gy, respectively. With regard to SREs, spinal cord compression and pathological fracture were not seen at treated sites after radiotherapy. On the other hand, there were three patients (8.6%) with pathological fracture and one patient (2.8%) with spinal cord compression in nontreated sites after radiotherapy.

## 4. Discussion

 Prostate cancer is the most frequently diagnosed cancer and is second only to lung cancer as the leading cause of cancer-related deaths among in the USA. In Japan, it is estimated that the incidence and mortality cases for prostate cancer will increase 3-fold by 2020 compared with 2000. Previous studies showed that independent prognostic variables for survival among patients with prostate cancer were patient age, time to androgen-independent disease, the extent of metastatic disease, and number of metastases on bone scan [14]. Several studies have focused on quantifying or stratifying risk according to the extent of bone involvement and the number of metastatic sites of prostate cancer [3, 15–17]. They have shown that the number of metastatic lesions is a powerful prognostic indicator of the outcome in metastatic disease. Among these studies, Soloway et al. [3] reported that a scale based on a count of the number of metastatic bone lesions on bone scan was predictive when ≤5 (EOD I) or >20 (EOD IV) lesions were present. On the basis of this result, we grouped our prostate cancer patients with bone metastases likewise and applied radiotherapy to metastatic bone disease in EOD I cases (i.e., oligometastases and oligo-recurrence of bone in prostate cancer) regardless of the presence of the bone pain. Results of this study revealed that the 3-year overall survival rate after radiotherapy to oligometastases or oligo-recurrence of bone was 77.2% in prostate cancer. To our knowledge, no previous study has examined overall survival in this patient population. Although the widely accepted treatment for patients with metastatic prostate cancer is hormonal therapy, we should manage oligometastases, oligo-recurrence, and polymetastases separately because of their difference in prognosis. Hellman and Weichselbaum [7] reported that local therapy such as radiotherapy and surgery for one or several distant metastatic sites could be efficacious for survival in patients with oligometastases. Niibe et al. [8] and Niibe and Hayakawa [9] also proposed oligo-recurrence, a more strictly defined type of oligometastases, in which one or several metastatic or recurrent lesions occur with the controlled primary lesions. They suggest that the local treatment of the metastatic or recurrent lesions could improve prognosis. Many studies have been performed along these lines [6–10]. Niibe et al. [8] also indicated that high-dose radiotherapy for bone metastases could contribute to patient survival in breast cancer. In the current study, because patient baseline characteristics were different between groups receiving a total radiation dose of ≥40 Gy or <40 Gy and there is few events on survival in each group, usual multivariable analysis could not be performed without propensity score. Therefore, radiotherapy for oligometastases and oligo-recurrence of bone in patients with prostate cancer is worth prospective testing as an approach to improving survival.

 The Radiation Therapy Oncology Group (RTOG) has previously studied various treatment fraction regimens for palliation of bone metastases. The RTOG 9714 study, a recent phase III trial centered on prostate cancer and breast cancer with osseous metastases, revealed 8 Gy per single fraction was equal to 30 Gy in 10 fractions for the pain relief of osseous metastases at 3 months after irradiation [18]. However, this study evaluation point for pain relief is very early, at 3 months after radiotherapy. This is not appropriate appreciation for oligometastases and oligo-recurrence because of long-term survival. Niibe et al. reported high-dose radiation contributed to long-term pain relief in breast cancer [8]. Milano et al. also reported high-dose stereotactic body radiotherapy for bone oligometastases, oligo-recurrence was efficacious [19]. Moreover, other investigation in the same population demonstrated that the retreatment rate was significantly higher in the 8 Gy arm (18%) than in the 30 Gy arm (9%) [20]. 

In Japan, longer courses of radiotherapy with higher total doses of radiation remain the most commonly used, typically with a regimen of 30–40 Gy given in 10–20 treatment sessions. While conventional radiotherapy was used in this study, the results reveal a median duration of pain relief of 12 months, with approximately half of the patients experiencing relapsed bone pain. The bone pain trial which include 34% of prostate cancer patients in patient population showed 40% of pain relapse at 12 months [18]. Although those patient characteristics are different from our study, we considered our result in duration of pain relief is comparable with that study. However, these results indicate that conventional radiotherapy alone for pain relief may be inadequate for oligometastases and oligo-recurrence of bone in prostate cancer. Consequently, for the management of bone pain in patients with prostate cancer, we should consider altering the radiation dose or fraction using high-dose SBRT combining it with treatments such as systemic chemotherapy, zoledronic acid, and painkiller. Punglia et al. [21] reported that as improving systemic therapy, local therapy got survival benefit dramatically. Niibe and Hayakawa [9] also reported the significance of systemic therapy for oligometastases and oligo-recurrence treated by local therapy.

 For patients without bone pain in this study, the main purpose of radiotherapy was prevention of SREs, including pathological fracture and spinal cord compression. The current study demonstrated that the complications were not seen in treated sites; however, three patients experienced pathological fracture and one patient had spinal cord compression in a nontreated site after radiotherapy. These results indicate that radiotherapy for metastatic bone disease may potentially decrease the incidence of SREs in treated sites. Both pathological fractures and spinal cord compression with neurologic deficit negatively affect quality of life [22]. Moreover neurologic recovery is unlikely if spinal compression is not relieved within 24–48 hours [23]. Therefore, efforts have recently been made to predict sites of fracture and to prevent the occurrence of a fracture by prophylactic therapy, which includes radiotherapy [24–26]. 

 Our study has several limitations. Because it is retrospective, patient populations differ between total radiation dose received (≥40 Gy and <40 Gy). There was also no control group, that is, one that did not receive radiotherapy. Therefore, in the future, a large prospective study is required to investigate the actual benefits, including overall survival associated with radiotherapy for oligometastases and oligo-recurrence of bone in prostate cancer.

## Figures and Tables

**Table 1:** Patient characteristics (35 patients).

Variables	<40 Gy (*n* = 14)	≥40 Gy (*n* = 21)	Total (*n* = 35)	*P* value*
Age	72 (66–85)	70 (55–93)	71.5 (55–93)^†^	0.206
Baseline PSA (ng/mL)	72.0 (0.3–964)^†^	11.0 (0.1–142)^†^	34.0 (0.1–964)^†^	0.047
ECOG PS				
0–1	8	21	29 (82.9%)	0.002
≥2	6	0	6 (17.1%)	
No. of bone metastases	3 (1–5)^†^	2 (1–5)^†^	2 (1–5)^†^	0.218
CRPC	5 (35.7%)	2 (9.5%)	7 (20%)	0.090
Pain				
Yes	9 (64.3%)	7 (33.3%)	16 (45.7%)	0.094
Spinal cord compression				
Yes	2 (14.3%)	0	2 (5.7%)	0.153
Pathologic fracture				
Yes	4 (28.6%)	1 (4.8%)	4 (14.3%)	0.134
Oligostatus				
oligo-recurrence group	2 (14.3%)	16 (76.2%)	18 (51.4%)	0.000

Abbreviations. PSA: prostate-specific antigen; ECOG PS: Eastern Cooperative Oncology Group performance status; CRPC: castration-resistant prostate cancer.

^†^Median (range).

^∗^Significance of difference between groups determined by chi-square test or Fisher exact test, as appropriate. *P* < 0.05 considered significant.

**Table 2:** Treatment characteristics.

Variables	Total *n* = 35
Total radiation dose (Gy)	40 (30–50)^†^
Biological effective dose (Gy_3_)	67 (50–92)^†^
Reasons for radiotherapy	
Pain	16 (45.7%)
Spinal cord compression	2 (5.7%)
Prevention for SREs	17 (48.6%)
Treatment site	
Spine	15 (42.9%)
Femur	17 (48.6%)
Pelvis/hip	3 (8.6%)
Sternum	1 (2.8%)
Ribs	2 (5.7%)
Overall treatment time (days)	28 (12–43)^†^

Abbreviations. SREs: skeletal-related events.

^†^Median (range).

**Table 3:** Treatment outcomes.

Variables		No. of patients (%)
	Short-term response	14 (87.5)
	No change	2 (12.5)
Pain relief (*n* = 16)	Progressive disease	0
	Long-term progression	5 (31.3)
	Time to progression (months)	9 (5–15)^†^

Incidence of SREs after radiotherapy (*n* = 35)	Pathologic fracture	
	Treatment site	0
	Nontreatment site	3 (8.6)
	Spinal cord compression	
	Treatment site	0
	Nontreatment site	1 (2.8)

Abbreviations. SREs: skeletal-related events.

^†^Median (range).

**Table 4:** Univariable and multivariable analysis for the effect of radiotherapy on survival.

Factors	Univariable analysis			Multivariable analysis		
	HR	95% CI	*P* value*	HR	95% CI	*P* value*
RTX (≥40 Gy versus <40 Gy)	0.231	0.067–0.798	0.021	0.630	0.098–4.285	0.637
Propensity score^†^	n/d	n/d	n/d	0.300	0.024–3.763	0.351

Abbreviations. HR: hazard ratio; n/d: not done.

^∗^Analyses were performed using Cox proportional hazard regression.

^†^Multivariable model indicates adjusted effect of RTX by applying propensity score which is a conditional probability of receiving RTX (≥40 Gy) given by other factors including age, baseline PSA, performance status, castration-resistant prostate cancer, and oligostatus.
